# Heart rate-defined sustained attention in infants at risk for autism

**DOI:** 10.1186/s11689-018-9224-2

**Published:** 2018-02-13

**Authors:** Bridgette L. Tonnsen, John E. Richards, Jane E. Roberts

**Affiliations:** 1 Department of Psychological Sciences, Purdue University, 703 3rd Street, West Lafayette, IN 47906 USA; 2 Department of Psychology, University of South Carolina, 1512 Pendleton Street, Columbia, SC 29208 USA

**Keywords:** Autism, Sustained attention, Heart activity, Infant siblings, ADOS

## Abstract

**Background:**

Although aberrant visual attention has been identified in infants at high familial risk for autism, the developmental emergence of atypical attention remains unclear. Integrating biological measures of attention into prospective high-risk infant studies may inform more nuanced developmental trajectories, clarifying the onset and course of atypical attention and potentially advancing early screening or treatment protocols. Heart rate-defined sustained attention (HRDSA) is a well-validated biological measure of attentional engagement that, in non-clinical infant populations, provides incremental information about attentional engagement beyond looking behaviors alone. The present study aimed to examine the characteristics and clinical correlates of HRDSA in high-risk infants, informing whether HRDSA may operate as a promising biological measure of attention and clinical symptoms in this population.

**Methods:**

We examined age-related patterns of HRDSA during a passive looking task in 5- to 14-month-old high-risk infant siblings of children with autism (*n* = 21) compared to low-risk controls (*n* = 21), with most participants contributing multiple assessments. Emergent autism features were measured using the Autism Diagnostic Observation Schedule at 24 months. Primary dependent variables included the proportion of time in behavioral attention, proportion of time in HRDSA, and average heart rate deceleration during HRDSA. For each variable, we used nested multilevel models to examine whether attention differed by group, as well as whether attention predicted emergent autism features among high-risk infant siblings.

**Results:**

As expected, HRDSA served as a global biological measure of attention in high-risk infants, predicting greater variability in group risk status than behavioral looking alone. Among high-risk infants, more severe ASD features were also associated with increasingly shallow heart rate deceleration during HRDSA across development, suggesting abnormal qualities of HRDSA may inform individual differences within this population.

**Conclusions:**

These preliminary findings provide initial evidence that HRDSA may offer a sensitive, affordable, and portable biological measure of attention that may enhance understanding of atypical attention in high-risk infants. Using this method, we also provide initial evidence that atypical patterns of heart activity previously reported in children and adults with autism may emerge in the first year of life, warranting further study of how HRDSA may specifically inform attention profiles in ASD.

## Background

Visual attention is one of the most robust early indicators of autism spectrum disorder (ASD), with higher rates of atypical features identified prior to age 12 months in infants later diagnosed with ASD [1, 2]. The majority of studies of early attention in ASD have been conducted with “high familial risk” (HFR) infant siblings of children with ASD who exhibit nearly 20 times the risk of ASD diagnoses than the general population [3]. Although a number of abnormal attention processes in HFR infants have been identified as potential “red flags” for ASD, the emergence and mechanisms of these behaviors remain unclear. Integrating biological measures of attention into prospective HFR infant studies may inform more nuanced developmental trajectories, clarifying the onset and course of atypical attention in HFR infants and potentially advancing early screening or treatment protocols. In infants, decelerations in heart rate offer one such biological measure that can be used to measure the presence and quality of “sustained attention,” the process whereby an infant exerts cognitive resources to process a stimulus [4–8]. Indeed, because heart rate deceleration has been shown to provide a more sensitive metric of sustained attention than behavioral looking alone [9], this method has been successfully applied to measure attention-related outcomes in a number of recent clinical infant nutrition studies [10, 11]. As such, it is possible that heart rate-defined sustained attention (HRDSA) may be similarly applied as a biological measure to more sensitively index attention in HFR infants. However, given self-regulatory deficits commonly reported in ASD [12, 13], it is unclear whether HRDSA will present similarly in HFR infants and previously studied low-risk populations. Thus, the present study aims to preliminarily characterize the nature and clinical correlates of HRDSA in HFR infants, informing whether HRDSA may operate as a promising biological measure of sustained attention and relevant clinical features—such as emergent ASD features—in this population.

### Heart rate-defined sustained attention

It is well-established that infants’ physical look durations do not cleanly map onto attention, as infants will often continue looking when they are no longer cognitively engaged in stimulus processing [14]. Thus for over five decades, heart rate-defined sustained attention has been integrated into developmental research as a well-validated, low-cost, and non-invasive biological measure of attentional engagement [15]. When the brain’s arousal system is activated, cardioinhibitory centers initiate parasympathetic processes to slow heart rate, producing heart rate decelerations that quantify qualities of stimulus engagement that cannot be measured using overt looking patterns alone [4–7]. Richards and colleagues have quantified three primary attention phases that occur when infants are physically looking toward a stimulus: orienting, sustained attention, and attention termination [4, 7, 16–18]. Heart rate-defined sustained attention (HRDSA) is indexed by maintenance of decelerated heart rate, reflecting the exertion of additional cognitive resources to process a stimulus. Indeed, infants in periods of HRDSA are less distractible across a variety of tasks, including computerized orienting paradigms [5, 19, 20] and toy play activities [21, 22]. HRDSA is also associated with enhanced neural responses and more accurate performance on behavioral learning tasks [19, 23–25], supporting HRDSA as a valid measure of attention in typically developing infants.

### HRDSA in special populations

Few studies have examined heart activity in HFR infants, and it is possible that HRDSA may present differently in HFR given the well-established autonomic abnormalities associated with ASD (see [12, 13] for review). A number of studies of older individuals with ASD have generally suggested faster overall heart rate in ASD [26–28] and difficulties modulating arousal when task demands change [29–31], although inconsistent findings are common [13]. Some evidence suggests that inconsistencies may reflect the broad, heterogeneous nature of ASD symptoms, as studies specifically focused on social communication deficits within ASD—as opposed to broader ASD status—have generally reported significant associations between ASD and physiological arousal [13]. In addition, it is possible that developmental factors complicate interpretation across studies. In a recent study, HFR infants exhibited slower heart rate (HR) at 3, 6, 9, and 12 months of age relative to low rate (LR) controls, conflicting with findings of hyperarousal in older individuals with ASD [13]. Notably, this pattern of slower HR in infants at high risk for ASD parallels previous findings in children with fragile X syndrome (FXS), a single-gene disorder highly associated with ASD. Specifically, infants with FXS who later exhibit high ASD features display slower HR in infancy (< 12 months) yet faster HR in the toddler and preschool period (12–40 months), relative to infants with FXS and low ASD features [32]. Given the complex, heterogeneous symptom profiles and developmental factors associated with ASD, further work is needed to characterize associations between heart activity and symptom profiles, particularly in early childhood.

Similarly, few studies have examined heart rate decelerations as a specific index of attention in ASD, and existing studies have used variable methods that preclude generalizable conclusions about the nature of HRDSA in ASD. Corona and colleagues examined behavioral responses and heart activity of 3- to 5-year-old children with ASD who viewed an examiner pretending to hurt herself, then displaying either an intense distress response or neutral affect [33]. Behaviorally, children with ASD and comparison children with intellectual disability both looked more toward the examiner displaying distress, although the ASD group exhibited atypical qualities of social attention, such as fewer looks toward the examiner’s face. However, only the control group exhibited decelerations in heart rate when examining the examiner in distress, potentially indicating blunted HRDSA toward the emotional stimulus in the ASD group. In contrast, Louwerse and colleagues identified typical heart rate decelerations in adolescents with ASD relative to non-ASD controls during a picture viewing task, with both groups exhibiting larger heart rate decelerations to unpleasant versus neutral stimuli [34]. Notably, both studies focused on heart rate decelerations as a metric of sustained attention in response to emotion-focused stimuli, rather than examining how HRDSA may inform general sustained attention in ASD. Thus, it remains unclear how HRDSA may inform general sustained attention and stimulus processing in ASD.

More recent work has begun expanding studies of HRDSA to infants at risk for ASD. In a longitudinal pilot study of 9- to 18-month infants with FXS (*n* = 13) and 12-month controls (*n* = 10), infants with FXS generally exhibited shallower heart rate decelerations and lower heart rate variability during a visual attention task [22]. However, despite infants with FXS displaying shallower decelerations at the group level, higher ASD symptoms were marginally associated with deeper heart rate decelerations and greater behavioral looking within the FXS group. Thus, despite an overall profile of physiological dysregulation in FXS, deeper heart rate decelerations converged with enhanced visual attention, similar to patterns observed in typically developing infant samples (e.g., [5, 19]). More recently, atypical patterns of heart rate decelerations were identified in HFR infants relative to low familial risk (LFR) controls from 3 to 12 months of age in response to speech stimuli [35]. Specifically, HFR infants exhibited atypically shallow heart rate decelerations across development, increasingly deviating from LR controls between 3 and 12 months of age, suggesting reduced social orienting during time. However, heart rate decelerations were not examined in relation to ASD outcomes; thus, it remains unclear whether atypically shallow decelerations observed in HFR infants are driven by the subset of HFR infants with later ASD. Together, these preliminary findings suggest that HRDSA may provide novel information about attention in infants at risk for ASD, although differences across stimuli, risk group, and availability of ASD outcome data complicate interpretations. For example, when HRDSA is used to measure social orienting (e.g., [35]), which is reduced in ASD, ASD risk may be associated with shallower HRDSA. In contrast, when HRDSA is used to measure responses to non-social visual stimuli (e.g., [22]), which are often reported to be atypically enhanced in ASD, ASD risk may be associated with increased HRDSA. Alternately, it is possible that differences in HRDSA patterns may be driven by biological or genetic factors, with FXS and HFR status mapping on to different patterns of physiological features. Given the limited work in this area, additional studies are needed to characterize the profiles of HRDSA in special populations, with particular attention toward intersecting developmental, clinical, and biological factors.

### HRDSA and visual attention in HFR infants

Although studies of HFR infants have critically advanced understanding of the emergence and early symptoms of ASD in infants, a major challenge to this work is the vast heterogeneity in individual profiles and features that present in infants later diagnosed with ASD. Biological measures such as HRDSA may improve HFR infant research by supporting more precise measurement of individual differences, reducing error and increasing power in HFR infant attention studies. Indeed, although abnormal cognitive and social attentional features have been observed in HFR infants and have been the topic of several reviews [36, 37], attention in HFR infants remains a complex topic, in part due to the diverse conceptualization and measurement of attention in the extant HFR infant literature. It is possible that physiologically derived indices of sustained attention could advance HFR infant attention research by providing information about the quality of attentional responses, particularly when looking behaviors are a primary variable of interest.

Relevant to the present study, a number of studies suggest abnormal attentional patterns in HFR infants across a variety of visual attention tasks, with particularly atypical profiles among infants with later ASD features. For example, HFR infants who later meet ASD diagnostic criteria exhibit longer saccadic latencies to disengage attention from competing stimuli [2, 38] and enhanced visual search performance [39]. In addition, studies focused on more socially salient tasks have demonstrated reduced attention-related behaviors in HFR infants, with later ASD features associated with reduced attention toward social scenes [40], people within a social scene [40], and a person’s eyes within a social scene [41] by 6 months of age. Fewer studies have examined sustained attention specifically in HFR infants, with one recent study suggesting disrupted social sustained attention in HFR infants who later meet criteria with ASD (*n* = 9) who exhibited shorter peak looks on social trials of a habituation task compared to HFR infants who did not meet ASD criteria (*n* = 49), although group differences were no longer significant at 12 months [42]. Limited work in older individuals is similarly complex, with some studies reporting reduced sustained attention in preschoolers with ASD [43], and others suggesting intact sustained attention in adolescents with high-functioning autism [44]. Reflecting this complexity, no single attentional profile established in either HFR infants in general or the subgroup of HFR infants who later meet criteria for ASD [45].

Improving the quality and depth of attention measurement during visual attention tasks, such as through the integration of HRDSA, may help clarify the scope, emergence, and mechanisms of abnormal attention in this population. At the phenotypic level, examining HRDSA may further inform whether sustained attention is disrupted in HFR infants, particularly those with later ASD features, building on a single study that has explicitly addressed this topic to date [42]. Methodologically, HRDSA could also be used to more sensitively capture the quality of attention engagement among HFR infants, as several recent studies have identified group differences in HRDSA despite similar behavioral sustained attention, such as in the context of habituation tasks [9] and nutritional clinical trials [11]. At the analytic level, HRDSA may also be used to reduce error in visual attention experiments by enabling researchers to isolate trials in which infants are physiologically engaged in an attention task. For example, typically developing infants exhibit more efficient stimulus processing and greater recognition memory when presented stimuli during HRDSA versus non-HRDSA phases [46]. Thus, researchers may control for HRDSA when examining condition effects or alternatively design experiments to selectively administer trials once a period of HRDSA has been achieved [21, 47]. Although other neuroimaging methods such as electroencephalogram (EEG) have similarly yielded more sensitive information about visual attention in HFR infants compared to behavioral methods [48, 49], HRDSA is a particularly attractive method given the relative ease, affordability, and portability of collecting infant heart rate data relative to more invasive or expensive neuroimaging techniques.

### The present study

Heart-rate defined sustained attention is a promising biological measure for measuring and tracking the development of attention in HFR infants given both the popularity of this method in typical developmental literature and its emergent promise in characterizing phenotypes in infants with FXS [22]. To test whether HRDSA may similarly inform attentional development in HFR infants, the present study examined levels and age-related changes in HRDSA in a prospective cohort of HFR infants and low-risk controls during a passive looking task. Although the focus of the present study is on HRDSA specifically, we expected age-related differences in task performance across groups, with LFR controls exhibiting reduced behavioral attention over time [50]. We also expected the task to solicit greater behavioral attention in HFR versus LFR infants, with increasingly distinct profiles between 6 and 12 months, based on previous findings that HFR infants with greater ASD features exhibit increasingly poorer disengagement from visual stimuli [1, 2] and neural changes predictive of ASD [51] between 6 and 12 months. In the context of these expected behavioral patterns, our goals were to characterize profiles of HRDSA in HFR infants relative to LFR infants, controlling for behavioral attention, as well as determine whether HRDSA corresponded with indices of emergent ASD features in the HFR group. This approach contributed to our overall aim of determining whether HRDSA may serve as a sensitive biological measure of sustained attention in HFR infants.

Given the exploratory nature of this study, we aimed to test two distinct hypotheses regarding the nature of HRDSA in HFR infants. The first hypothesis was that HFR infants would display greater HRDSA, indexed by greater proportion of time in HRDSA and deeper heart rate decelerations during HRDSA, and that these patterns would be most atypical in infants who later exhibited more severe ASD features. This hypothesis reflects a global assumption that HRDSA would relate to behavioral sustained attention similarly across HFR and LFR groups, with greater HRDSA paralleling greater behavioral attention, similar to previous studies of typically developing infants [5, 19]. Indeed, older children with ASD have been shown to exhibit typical increases in HRDSA toward emotional stimuli [34] and decreased HRDSA that parallels reduced quality of social attention [33], suggesting HRDSA may converge with behavioral responses in this population. Similarly, greater heart rate decelerations have been preliminarily associated with greater ASD symptoms in infants with FXS, despite atypically shallow decelerations in FXS relative to controls [22]. Thus, this first hypothesis posited that HRDSA in HFR infants would operate in a qualitatively similar manner to LFR infant studies, potentially advancing the sensitivity of sustained attention measurement in HFR infant studies.

Our alternative hypothesis was that HFR infants, particularly those with greater emergent ASD features, would display enhanced behavioral attention yet *shallower* heart rate decelerations during HRDSA, similar to enhanced behavioral attention and shallower heart rate decelerations previously observed in fragile X syndrome [22]. This hypothesis would suggest that in Corona and colleagues’ previous study, adolescents with ASD may have failed to exhibit typical heart rate decelerations across conditions due to a dysregulated sustained attention response, rather than a blunted emotional response to the examiner’s distress. This hypothesis is also consistent with previous studies in which individuals with ASD exhibit hyperarousal and difficulty modulating arousal across changing task conditions [29–31], as well as evidence that increasingly faster heart rate across toddlerhood predicts ASD symptoms in FXS [32]. In other words, it is possible that even during infancy, HFR status may be associated with dysregulated sustained attention responses that manifest in blunted HRDSA, potentially in concert with emergent ASD features in a subset of infants. If upheld, the implications of this hypothesis are that HRDSA may provide insight into the mechanisms sustaining atypical attention profiles in HFR infants, rather than solely advancing sustained attention measurement in this group.

## Methods

### Participants

Participants were drawn from an ongoing study of early development in HFR infants. The final sample included 42 infants (17 males, 4 females per group) assessed between 5 and 14 months of age across 73 observations. Table 1 includes participant characteristics. Infants were born full term (37 weeks, > 2000 g) and lived with their biological mother due to the broader study focus on familial and genetic risk factors for ASD. HFR infants were full biological siblings of a child with a documented ASD diagnosis, verified using community medical diagnostic reports. Low-risk (LFR) controls were recruited from the community, were required to fall in the “low risk” range on the Autism Observation Scale for Infants [52] at 12 months, and did not have a family history of ASD. Infants entered the study at variable ages, enrolling between 4.5 and 10.5 months and completing up to three assessments approximately 3 months apart. In addition to the 73 assessments analyzed for these infants, 25 additional assessments (13 HFR, 12 LFR) were conducted but excluded due to excessive error in heart activity data (*n* = 15), technical difficulties (*n* = 9), and the infant refusing to sit (*n* = 1). Groups exhibited similar numbers of participants with one (HFR *n* = 10, LFR *n* = 10), two (HFR *n* = 6, LFR *n* = 7), and three assessments (HFR *n* = 5, LFR *n* = 4).

**Table 1:** Descriptions of primary variables at both individual and assessment levels

	High familial risk (*n* = 21)					Low familial risk (*n* = 21)				
	*n*	$\overline{X}$	SD	min	max	*n*	$\overline{X}$	SD	min	max
Individual-level variables										
*n* assessments	21	1.76	0.83	1	3	21	1.71	0.78	1	3
MSEL Early Learning Score	21	97.62	16.26	60	137	21	102.86	10.96	80	117
ADOS Severity Score	20	3.95	2.76	1	10	–	–	–	–	–
Assessment-level variables										
Age in months	37	9.80	2.43	5.98	13.41	36	10.23	2.23	5.69	13.74
Proportion time inattentive	37	0.40	0.25	0.08	0.95	36	0.43	0.22	0.03	0.81
Proportion time in HRDSA	37	0.30	0.21	0.00	0.92	36	0.30	0.21	0.06	0.95
IBI change during HRDSA	34	33.38	23.53	3.96	110.47	36	36.02	22.52	3.56	130.15

Individual-level variables are measured on one occasion per individual, and assessment-level variables are collapsed across all assessments and individuals in each group

*ADOS* Autism Diagnostic Observation Schedule, *HRDSA* heart rate-defined sustained attention, *IBI* interbeat interval, *MSEL* Mullen Scales of Early Learning

### Procedure and measures

Procedures were approved by the Institutional Review Board. Parents provided consent prior to the onset of the study and were compensated for participation. To minimize family travel, attention assessments alternated between laboratory and home environments. Experimental setup was standardized across settings using a portable, nonreflective black felt shield. As part of a larger battery, each assessment included a passive viewing attention task and additional developmental and clinical evaluations. The attention task was attempted at the beginning of each assessment and was later re-attempted if the infant was fussy or would not comply. The present study includes behavioral and heart activity data from all assessments, as well as developmental testing conducted around 12 months of age. Follow-up ASD diagnostic assessments were conducted in the laboratory around 24 months.

#### Passive viewing task

During each assessment, participants’ looking behavior and heart activity were measured while they viewed an engaging 135-s *Baby Einstein* children’s video depicting animal puppets, moving toys, and classical music. Participants were seated in a darkened room, 10 in. away from an “11 × 24” LCD monitor. Two video cameras simultaneously recorded stimuli and participants’ faces to capture whether infants were looking at the screen. Electrocardiogram signal (ECG) was collected using Alive heart monitors (Alive Corporation, Gold Coast, Australia), and signal was transmitted live to a laptop via Bluetooth. If the child did not look at the video voluntarily, the examiner provided up to three prompts to re-engage the child, after which the child was permitted to look toward or away from the screen.

#### Autism diagnostic symptoms

The Autism Diagnostic Observation Schedule—Toddler Module (ADOS-T) [53] is a well-established diagnostic measure of ASD symptom severity appropriate for children ages 12 to 30 months. The ADOS-T was administered when children were approximately 24 months old by staff trained to research-reliability standards for both administrating and scoring. One participant was not able to return for ADOS testing until age 43 months and was administered the ADOS-2 Module 2, consistent with standardized guidelines based on age and verbal ability. We computed continuous severity scores (CSS) using established algorithms [54] (range 1–10). Of the 20 HFR infants with ADOS data, 11 received overall total scores in the “little to no concern” (ADOS-T) or “nonspectrum” (ADOS-2) range, 6 in the “mild-to-moderate” range, and 3 in the “moderate to severe” range. The remaining infant was lost to follow-up and did not complete ADOS testing.

#### Developmental ability

The Mullen Scales of Early Learning (MSEL) [55] is a standardized measure of cognitive development for children under 68 months. Participants’ Early Learning Composite score from the 12-month assessment was modeled as a covariate in a subset of analyses.

### Quantification of behavioral and heart activity variables

Behavioral data were integrated into Observer XT 10.1 software [56] for offline coding. Proportion of time looking toward the screen (“behavioral looking”) was calculated for each participant. Interrater agreement was 83 across 20% of randomly sampled assessments.

Heart activity artifact editing was completed by a coder trained to research reliability through a standardized training sequence supervised by the Brain-Body Center staff [57]. Files that required coders to edit greater than 5% of interbeat intervals (IBI) were excluded from analyses (*n* = 15; 6 HFR infant, 9 LFR), consistent with previous studies of HRDSA in ASD [33]. Data were edited for artifacts and analyzed using the CardioEdit and CardioBatch programs [57]. Next, duration and magnitude of heart rate deceleration during HRDSA were analyzed using mean-change algorithms [4] in SAS 9.3. For each period of behavioral looking, HRDSA was characterized by comparing IBI to baseline, defined as the median IBI of 5 beats preceding gaze toward the screen. Baseline values were reset each time the participant looked away from the screen for more than 1.5 s. The onset of HRDSA was indexed by 5 successive beats with longer IBIs than baseline, and HRDSA terminated after 5 successive beats with IBIs shorter than baseline. HRDSA was quantified using two primary variables: proportion of time in HRDSA and average heart rate deceleration during HRDSA, quantified as average change in IBI from baseline. Three HFR infants did not exhibit HRDSA phases, likely related to spending low proportions of time looking at the screen (13–20% looking time versus average of 60% among HFR infants; Table 1). These infants displayed low ASD symptoms (ADOS CSS: 3, 1, 3).

### Analytic plan

Analyses were conducted using SAS 9.4 (Apex, NC) with α set to less than 0.05. Data were evaluated for analytic assumptions and outliers prior to analyses. Groups did not differ in number of assessments per participant, *F*(1, 40) = 0.04, *p* = .85, mean chronological age across assessments, *F*(1, 71) = 0.62, *p* = .44, or developmental level at outcome *F*(1, 40) = 1.50, *p* = .23. Developmental level did not correlate with any dependent variable across the sample or within either group. However, developmental level was lower among HFR infants who exceeded the ADOS diagnostic threshold (Wilcoxon *Z* = 2.48, *p* = .02); thus, ADOS models were repeated with developmental level covaried to ensure effects were not driven by lower developmental level. The following variables which were log-transformed due to non-normality: proportion looking time, proportion time in HRDSA, average IBI deceleration during HRDSA, and ADOS CSS.

Analyses focused on three primary dependent variables: proportion of time in behavioral attention, proportion of time in HRDSA, and average depth of heart rate deceleration during HRDSA. To test for the incremental effects of HRDSA over behavioral looking alone, we controlled for the main effect of behavioral attention (centered at the mean) and the interaction between behavioral attention and the relevant level 2 predictor (group or ADOS CSS) when conducting models examining HRDSA. Due to the nested structure of participant data, we analyzed levels and change in attention using mixed effects multilevel models. Preliminary ICC and model fit analyses indicated that data clustered within individuals; however, the association between dependent variables and age was relatively stable across individuals. Thus, only intercepts were modeled as random effects, and age effects are interpreted as age-related patterns that account for nesting within individuals, rather than differences in individual growth curves.

For our first step of analyses, we characterized general patterns of each variable across age using unconditional models. Next, we constructed a series of conditional models to test the hypotheses that HFR infants would be more behaviorally attentive and display greater HRDSA, indexed by greater proportion of time in HRDSA and deeper heart rate decelerations during HRDSA. These models tested the effect of group membership (level 2 predictor) on initial level and change of behavioral attention (proportion time looking toward screen) and heart rate-defined sustained attention (proportion time in HRDSA, average heart rate deceleration during HRDSA). Finally, we constructed a second set of conditional models to test whether among HFR infants, higher severity of ASD symptoms (ADOS CSS) would be associated with more abnormal behavioral attention and HRDSA. To do so, we repeated the multilevel analyses in the HFR infant group only, replacing group with ADOS CSS as the level 2 predictor. For each conditional model, we estimated proportion of variance explained by predictors using pseudo *R*^2^ [58].

## Results

### Cross-group differences in HRDSA

Preliminary unconditional models indicated that across the sample, each primary dependent variable (proportion of time in behavioral attention, proportion of time in HRDSA, average heart rate deceleration during HRDSA) was relatively stable across age. However, as expected, conditional models indicated group differences in behavioral attention, with the HFR group failing to exhibit typical decreases in attention across age. Fixed effects for these models are presented in Table 2.

**Table 2:** Fixed effects and standard errors of group membership and ASD symptoms on attention parameters

Group comparisons	*R* ^2^	Intercept	Age	Group	Group × age	% inattention	% inattention × Age
Proportion time inattentive	.19	0.32 (0.03)**	0.02 (0.01)*	0.03 (0.05)	− 0.04 (0.01)**	–	–
Proportion time in HRDSA	.54	0.29 (0.02)**	− 0.02 (0.01)*	− 0.04 (0.03)	0.02 (0.01)*	− 0.64 (0.08)**	0.02 (0.03)
IBI change during HRDSA	− .06	3.39 (0.14)**	0.04 (0.05)	− 0.17 (0.19)	− 0.02 (0.08)	− 0.60 (0.57)	− 0.16 (0.22)
Autism symptom severity (HFR infants only)	*R* ^2^	Intercept	Age	Autism	Autism × age	% inattention	% inattention × Age
Proportion time Inattentive	− .01	0.48 (0.10)**	− 0.004 (0.03)	− 0.07 (0.06)	− 0.01 (0.02)	–	–
Proportion time in HRDSA	.67	0.30 (0.06)**	0.02 (0.02)	− 0.03 (0.04)	− 0.02 (0.01)	− 0.81 (0.12)**	− 0.04 (0.05)
IBI change during HRDSA	.39	3.51 (0.35)**	0.24 (0.07)**	− 0.18 (0.22)	− 0.19 (0.05)**	− 0.56 (0.64)	− 0.52 (0.26)*

*p < .05, **p < .01

*HRDSA* heart rate-defined sustained attention, *IBI* interbeat interval

**Fig. 1:**
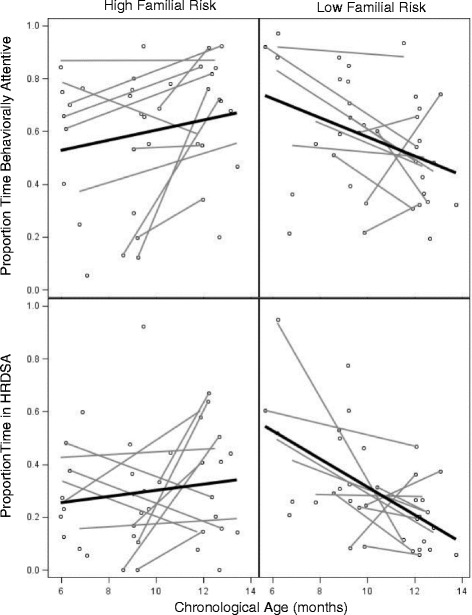
Group difference in behavioral and heart rate-defined attention across age. Note: gray = nested data for individuals with multiple assessments; black = group regression line

Controlling for behavioral attention, groups differed in the proportion of time spent in HRDSA, with combined predictors accounting for 54% of model variability. Figure 1 depicts this association, demonstrating individual data points (circles) and linear trajectories (gray lines) of behavioral and HRDSA across age, as well as average trajectories for each group across all data points (thick black lines). As this figure depicts, the LFR group exhibited age-related decreases in both behavioral attention and HRDSA across age, whereas the HFR infant group exhibited greater stability over time. Critically, proportion of time in HRDSA distinguished groups with behavioral attention controlled in the model. These data suggest that although both HRDSA and behavioral attention patterns distinguished groups, HRDSA was more sensitive to HFR group status than looking behavior alone.

**Fig. 2:**
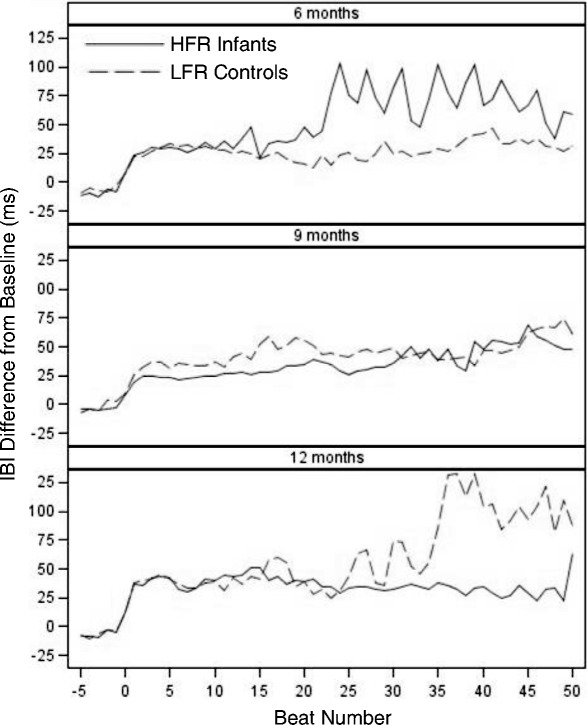
Average interbeat interval change across sustained attention by group and age. Note: To reflect group averages, phase data were truncated at 50 beats due to the low incidence of longer sustained attention phases. HFR high familial risk infant siblings, LFR low familial risk controls

Contrary to hypotheses, groups did not differ in average heart rate deceleration during HRDSA. Figure 2 depicts beat-by-beat interbeat interval (IBI) data for all participants’ HRDSA phases by group and age, truncated at 50 beats to reflect the majority of data. Participants were separated into age bins for display purposes only, as age was analyzed continuously. As this figure depicts, groups exhibit relatively similar trajectories of IBI change across HRDSA. Thus, groups were primarily distinguished by overall proportion of time in behavioral attention and HRDSA, with age-related decreases in in the LFR but not HFR infant group.

### Predictors of diagnostic symptom severity in HFR infants

**Fig. 3:**
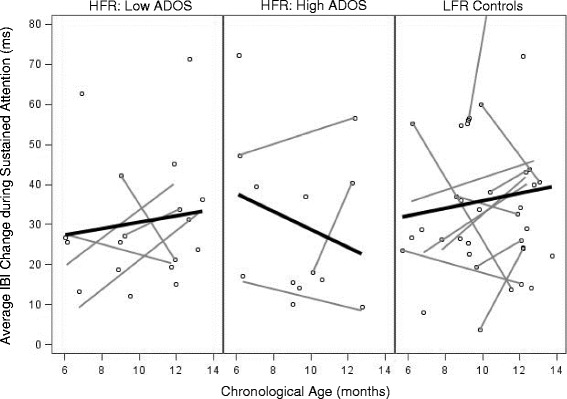
Average IBI change during HRDSA across age, separated by ADOS score. Note: Although data were analyzed continuously, “high” ADOS scores in the figure include those in the “mild-to-moderate” and “moderate-to-severe” ranges. LFR group values are displayed for reference. One LFR participant value not pictured to maintain graph scale (IBI change = 130.15 ms at age 12.03 months). Note: gray = nested data for individuals with multiple assessments; black = group regression line

We next examined the association between ASD features and attention within the HFR group only. Participants’ ADOS CSS were associated with abnormal age-related patterns of heart rate deceleration during HRDSA. As depicted in Fig. 3, average heart rate deceleration (quantified as change in IBI) was relatively stable in HFR infants with low ADOS scores, similar to general patterns in the LFR group. However, participants with higher ADOS scores generally exhibited reduced heart rate deceleration across age. This effect was maintained when overall cognitive abilities were covaried, (interaction B = − 0.19, SE = .05, *p* = .002), suggesting the association between heart rate deceleration and ADOS scores was not driven by lower cognitive abilities in children with higher ADOS scores. Thus, among HFR infants, higher ADOS scores were associated with increasingly blunted heart rate decelerations during HRDSA across age.

## Discussion

Integrating low-cost, accessible biological measures of attention in HFR infants may advance efforts to clarify the attentional phenotype of HFR infants, including potential predictors of later ASD features. Although abnormal attention and heart activity are well-documented in ASD and have been posited to relate to ASD emergence [13], the intersection of attention, heart activity, and autism in infancy is poorly understood. The present study applied a well-established biological measure of infant attention, HRDSA, to a prospective cohort of HFR and LFR infants during a passive viewing task. As expected, HRDSA served as a useful metric of attention in HFR infants, predicting greater variability in HFR status than behavioral looking alone. In addition, infants’ HRDSA quality, as measured by the depth of heart rate deceleration during HRDSA, was also sensitive to clinical features among HFR infants, with increasingly shallow HRDSA across age in children with more severe ASD features. These preliminary findings warrant further investigation of HRDSA in HFR infants, with particular focus on applications as a low-cost, accessible biological measure of sustained attention that, with further study and validation, may also inform clinical risk.

### HRDSA distinguished HFR infant status

Our first major finding was that proportion of time in HRDSA provided a more sensitive index of group status than behavioral looking alone. This finding generally supports HRDSA as a valid biological measure of sustained attention in HFR infants, paralleling previous studies in which HRDSA converged with behavioral engagement during social attention paradigms in children with ASD [33, 34]. Critically, our data also suggest that HRDSA provides enhanced sensitivity to group status compared to behavioral looking alone, with combined predictors including group status (HFR versus LFR) explaining 54% of variability in HRDSA compared to 19% in behavioral looking. The enhanced sensitivity of HRDSA to group status in HFR versus LFR infants converges with recent evidence that proportion of time in HRDSA is a more sensitive metric of treatment response than behavioral looking in infant nutrition clinical trials [11] and can be used to detect infant learning that is not obvious from looking behaviors [9]. Thus, the enhanced sensitivity of HRDSA to HFR infant status offers promise for future studies seeking to more sensitively index sustained attention in HFR infants.

Notably, the group differences we observed in sustained attention across HFR and LFR groups—indexed both behaviorally and autonomically—also advance the HFR infant literature. As we expected, HFR infants failed to exhibit typical decreases in behavioral attention and HRDSA observed in LFR controls, manifesting in increased attention among HFR infants at older ages. Behaviorally, these patterns parallel previous studies in which HFR infants, particularly those with later ASD features, display reduced visual engagement at 6 months [42], fail to reduce look durations between 6 and 12 months [42], and take longer to disengage attention from competing stimuli by 12 months of age [2, 59]. Thus, behavioral and autonomic indices from our study and others suggest atypical attention that is present in a subset of HFR infants by 6 months of age, well before the traditional age of ASD diagnosis.

### HRDSA predicted individual differences among HFR infants

Our second major finding was that among HFR infants, later ASD symptoms were uniquely predicted by increasingly shallow HRDSA across age, despite ASD symptoms remaining unrelated to overall time spent in behavioral attention or HRDSA. These data suggest that in addition to providing a valid index of sustained attention, HRDSA may be sensitive to *qualities* of attention engagement that are related to emergent ASD features not detectable by *quantities* of attention alone. From a theoretical perspective, these findings suggest that dysregulated HRDSA may be relevant to emergent ASD features in HFR infants. Although preliminary, this finding maps onto several relevant studies of heart activity in neurodevelopmental disorders. First, these patterns provide preliminary but novel evidence that autonomic dysfunction observed in children and adults with ASD [12, 13] may be detectable during the first year of life in a subset of HFR infants. Indeed, higher clinical risk was associated with relative hyperarousal at older assessments, similar to previous findings that ASD is associated with hyperarousal in children with idiopathic ASD [27] and fragile X syndrome [32]. Our findings also converge with a recent report that HFR infants display shallower heart rate decelerations across 6–12 months in response to speech stimuli, potentially reflecting reduced social orienting over time [35]. Interestingly, similar patterns of shallower HR decelerations during HRDSA have been reported in infants with FXS, although within this sample, deeper decelerations are marginally associated with higher ASD symptoms [22]. Together, these data suggest that qualities of HRDSA may provide insight into the mechanisms of abnormal attention in HFR infants who later meet criteria for ASD, although additional work is needed to replicate and further characterize these associations.

### Limitations and future directions

This preliminary study supports the need for further work examining HRDSA as a biological measure of attention in HFR infants. However, as with any biological measure relevant to ASD, further study of HRDSA should rigorously characterize the scope and limitations of applying HRDSA to HFR infant work, as well as carefully validate any future translational efforts [60]. It will be important to validate and replicate these findings in an expanded prospective HFR sample, attending to the specific onset of atypical HRDSA in HFR infants and patterns of change across development. This work is particularly important given a small number of HFR infants received elevated ADOS scores in our sample (*n* = 9), and our primary focus on ASD features continuously rather than a categorical diagnosis. It will also be important to clarify how methodological issues such as task design (e.g., passive versus active tasks) and stimulus types (e.g., computerized versus naturalistic; social versus nonsocial) may affect findings. Further work is also needed to clarify whether global arousal atypicalities and attention-specific impairments present as additive or multiplicative components of risk, as it is unclear whether abnormal HRDSA is a downstream effect of broader autonomic dysregulation reported in ASD. In addition, it will be important to establish long-term profiles and developmental cascades associated with abnormal heart activity in HFR infants, including establishing the specificity of these associations to ASD and the stability of these associations over time. Finally, future work should also examine the association between HRDSA and ASD in across multiple high-risk samples simultaneously. For example, comparing HRDSA in HFR infants and infants with FXS may clarify the specific correlates of ASD that span multiple groups, given ASD features were associated with decreased heart rate decelerations in our HFR sample versus increased decelerations in FXS [22]. This work is particularly important given the heterogeneous outcomes associated with HFR infant status [61] by providing a means to informing the specificity and generalizability of ASD precursors in infancy.

## Conclusions

The present study provides initial evidence that HRDSA may offer a sensitive, affordable, and portable biological measure of attention that may enhance understanding of the intersection of heart activity and attention in HFR infant samples. Using this method, we also provide initial evidence that atypical patterns of heart activity previously reported in children and adults with ASD may emerge in the first year of life among a subset of HFR infants, warranting further study of how HRDSA may specifically inform emergent ASD features. This work warrants further study of HRDSA as a potential biological measure of attention and clinical risk in HFR infants.

## Abbreviations

ADOS: Autism Diagnostic Observation Schedule
ASD: Autism spectrum disorder
HFR: High familial risk
HRDSA: Heart rate-defined sustained attention
IBI: Interbeat interval
LFR: Low familial risk
